# Surgical management of Diabetic foot ulcers: A Tanzanian university teaching hospital experience

**DOI:** 10.1186/1756-0500-4-365

**Published:** 2011-09-24

**Authors:** Phillipo L Chalya, Joseph B Mabula, Ramesh M Dass, Rodrick Kabangila, Hyasinta Jaka, Mabula D Mchembe, Johannes B Kataraihya, Nkinda Mbelenge, Japhet M Gilyoma

**Affiliations:** 1 Department of Surgery, Weill-Bugando University College of Health Sciences, Mwanza, Tanzania; 2 Department of Orthopaedics, Weill-Bugando University College of Health Sciences, Mwanza, Tanzania; 3 Department of Internal Medicine, Weill-Bugando University College of Health Sciences, Mwanza, Tanzania; 4 Department of Surgery, Muhimbili University of Health and Allied Sciences, Dar Es Salaam, Tanzania

**Keywords:** Diabetic foot ulcers, prevalence, pattern, surgical management, Tanzania

## Abstract

**Background:**

Diabetic foot ulcers (DFUs) pose a therapeutic challenge to surgeons, especially in developing countries where health care resources are limited and the vast majority of patients present to health facilities late with advanced foot ulcers. A prospective descriptive study was done at Bugando Medical Centre from February 2008 to January 2010 to describe our experience in the surgical management of DFUs in our local environment and compare with what is known in the literature.

**Findings:**

Of the total 4238 diabetic patients seen at BMC during the period under study, 136 (3.2%) patients had DFUs. Males outnumbered females by the ratio of 1.2:1. Their mean age was 54.32 years (ranged 21-72years). Thirty-eight (27.9%) patients were newly diagnosed diabetic patients. The majority of patients (95.5%) had type 2 diabetes mellitus. The mean duration of diabetes was 8.2 years while the duration of DFUs was 18.34 weeks. Fourteen (10.3%) patients had previous history of foot ulcers and six (4.4%) patients had previous amputations. The forefoot was commonly affected in 60.3% of cases. Neuropathic ulcers were the most common type of DFUs in 57.4% of cases. Wagner's stage 4 and 5 ulcers were the most prevalent at 29.4% and 23.5% respectively. The majority of patients (72.1%) were treated surgically. Lower limb amputation was the most common surgical procedure performed in 56.7% of cases. The complication rate was (33.5%) and surgical site infection was the most common complication (18.8%). Bacterial profile revealed polymicrobial pattern and *Staphylococcus aureus *was the most frequent microorganism isolated. All the microorganisms isolated showed high resistance to commonly used antibiotics except for Meropenem and imipenem, which were 100% sensitive each respectively. The mean hospital stay was 36.24 ± 12.62 days (ranged 18-128 days). Mortality rate was 13.2%.

**Conclusion:**

Diabetic foot ulceration constitutes a major source of morbidity and mortality among patients with diabetes mellitus at Bugando Medical Centre and is the leading cause of non-traumatic lower limb amputation. A multidisciplinary team approach targeting at good glycaemic control, education on foot care and appropriate footware, control of infection and early surgical intervention is required in order to reduce the morbidity and mortality associated with DFUs. Due to polymicrobial infection and antibiotic resistance, surgical intervention must be concerned.

## Background

Diabetic foot ulcers (DFUs) pose a major public health problem worldwide and contribute significantly to morbidity and mortality of patients with diabetes [1]. It is estimated that 15% to 20% of patients with diabetes will develop an ulcer on their foot at some point, and for many of these cases, the most appropriate treatment results in some form of surgery [2].

Diabetic foot ulcers are the most common cause of non-traumatic lower limb amputations in developing countries, and the risk of lower extremity amputation is 15 to 46 times higher in diabetics than in persons who do not have diabetes mellitus [3].

The prevalence of diabetic foot ulcers ranged between 1.0% and 4.1% in the United States, 4.6% in Kenya, and 20.4% in Netherlands [4-6]. Similarly, numerous hospital-based studies in Nigeria demonstrated that the prevalence of limb ulcerations was between 11.7% and 19.1% among individuals with diabetes in Nigeria [7,8]. The prevalence of DFUs among hospitalized patients with diabetes in Iran was 20% [9].

The burden of diabetic foot ulceration is heaviest in the resource-poor parts of the world where the incidence is high but sophisticated and efficient diagnostic, therapeutic and rehabilitative facilities are sparse. The challenge of management of DFUs in developing countries is that most patients with DFUs present to healthcare facilities late with advanced foot ulcers. The reasons for the late presentation include poor economic capabilities in cost shared healthcare systems, inadequate knowledge of self-care, socio-cultural reasons and poor and inadequate diabetes healthcare [5]. Studies in Tanzania have shown that surgical intervention of DFUs after the onset of gangrene may be too late to prevent death [10,11], therefore early presentation by patients and prompt surgical intervention during less severe rather than during later stages of an ulcer may improve patients outcome and reduce mortality rates [11].

There is paucity of published data on surgical management of DFUs in our environment as there is no local study which has been done in any hospital in Tanzania and Bugando Medical Centre in particular. This study was intended to describe our own experience in the surgical management of DFUs in our local setting, outlining the prevalence, pattern and treatment outcome of DFUs and compare our results with that reported in literature.

## Methods

### Study design and setting

This was a hospital based prospective study of all patients with diabetic foot ulcers seen in the surgical wards and at the surgical outpatient clinics of Bugando Medical Centre (BMC) over a two-year period from February 2008 to January 2010 inclusive. BMC is a tertiary care, consultant and teaching hospital for the Weill-Bugando University College of Health Sciences (WBUCHS). It has a bed capacity of 1000. BMC is one of the four largest referral hospitals in the country and serves as a referral centre for tertiary specialist care for a catchment population of approximately 13 million people from Mwanza, Mara, Kagera, Shinyanga, Tabora and Kigoma. Diabetic patients are first seen in the internal medicine department where screening for the foot at risk for ulceration is done, and only patients who are found to have active foot ulceration are presented to surgeons.

### Study subjects

All patients who presented to the surgical wards or surgical outpatient clinic with diabetic foot ulcers were consented for the study and those who met the inclusion criteria were consecutively enrolled into the study. Patients with healed foot ulceration were excluded from the study. Identification of patients with the foot at risk for ulceration was done in the medical wards or diabetic clinics and diabetic patients who were found to have active foot ulceration were referred to the surgical wards or surgical outpatient clinics for proper surgical management. Diabetic foot ulcer was operationally defined as a breach on the normal skin occurring as induration, ulceration or change of color on the foot for duration equal to or more than two weeks. A detailed history and physical examination was done and included the following: Patient's characteristics e.g. age, sex, area of residence, occupation, education level and presence of pre-morbid illness; Clinical characteristics including: duration of diabetes, types of diabetes (type I or II), duration of foot ulcer and patient's awareness of its presence, mode of treatment received, previous knowledge of foot care, previous history of healed foot ulcers, type of DFUs (neuropathic, ischemic, neuro-ischemic) and Wagner's classification; Operative characteristics included: type of operations performed and post- operative complications; Major lower limb amputation was defined as amputation at or proximal to the ankle joint whereas amputation distal to the ankle joint were termed as minor lower limb amputation. Outcome characteristics included: Length of hospital stay, mortality. Investigations including blood sugar profile, the glycated haemoglobin (HbAlc), renal functions, swabs from wound / ulcer and X-ray of foot carried out were also recorded. Assessment of glycaemic control was done by estimation of glycated haemoglobin (HbAlc). The glycated haemoglobin (HbAlc) was analyzed using the calorimetric end-point method on the IMx machine whose normal non-diabetic range is 4.4-6.4%HbAlc. The results were then reported in percentage graded as per assay test recommendation as:

HbAlc ≥ 7% good metabolic control

HbAlc 7-10% fair control

HbAlc ≤10% poor metabolic control

The diagnosis of surgical site infection was based on careful clinical examination (purulent discharge from the wound + signs of inflammation) and identification of micro-organisms from the area of the operative wound suspected of being infected.

The DFUs were graded according to Wagner's classification [12]. In order to describe the type of foot ulcers, both feet were examined for the presence or absence of peripheral sensation or pulses (dorsalis pedis and posterior tibial arterial pulses). Foot ulcers were categorized as ischemic when peripheral pulses were absent but the sensation was intact, neuropathic when sensation was absent but the peripheral pulses were intact and neuro-ischemic when both sensation and peripheral pulses were absent.

### Data collection and Statistical analysis

Data were collected using a designed questionnaire. The questionnaire was pre-tested before use to a small sample of 10 diabetic patients to determine whether the respondents have any difficulty in understanding the questionnaire and whether there are ambiguous or biased questions. Data collected were analyzed using SPSS computer software 15.0. Data were expressed in form of proportions and frequency tables for categorical variables. Means and standard deviation were used to summarize continuous variables. The test statistics used included student's t test and Chi squared test. The student's t test was used to test for differences between quantitative variables and Chi squared test was used to test for associations and comparisons of proportions. Significance was defined as a *p-*value of less than 0.05.

## Results

Of the total 4238 diabetic patients seen at Bugando Medical Centre during the study period, 136 patients (3.2%) had foot ulcers. Of these, 115 (84.6%) patients were hospitalized and the remaining 21 (15.4%) patients were treated as outpatients. Seventy-four patients (54.4%) were males and females were sixty-two (45.6%) with the male to female ratio of 1.2:1. Their mean ages was 54.32 ± 16.24 years (ranged from 21to 72 years. The modal age group was 51-60 years.

The majority of patients (98; 72.1%) came from the rural areas located a considerable distance from Mwanza City and most of them (97; 71.3%) had either primary or no formal education. Smoking habits and alcohol use was reported in 35.3% and 49.3% of patients respectively. Four patients (4.4%) had family history of diabetes mellitus (Table 1).

**Table 1:** Socio-demographic characteristics

Patient characteristics	Number of patients	Percentages
**Age (in years)**		
< 40	2	1.5
41-50	50	36.8
51-60	60	44.1
> 60	34	17.6
**Sex**		
Males	74	54.4
Females	62	45.6
**Area of residence**		
Rural	98	72.1
Urban	38	45.6
**Education**		
No formal education	46	33.8
Primary education	57	37.5
Secondary education	27	15.9
Tertiary education	12	8.8
**Premorbid illness**		
Present	12	8.8
Absent	124	91.2
**Smoking habits**		
Yes	48	35.3
No	88	64.7
**Alcohol use**		
Yes	67	49.3
No	69	50.7
**Family history of diabetes mellitus**		
Yes	6	4.4
No	130	95.6

Of the total 136 patients, 38 (27.9%) were newly diagnosed diabetic patients. The majority of patients (134, 95.5%) had type 2 diabetes mellitus. The median duration of diabetes was 8 years while the median duration of foot ulcers was 18 weeks. The majority of patients (64; 47.1%) presented between four weeks and 52 weeks of onset of an ulcer (median = 12 weeks). Fourteen patients (10.3%) had previous history of foot ulcers and six patients (4.4%) had previous amputations.

Seventy-nine (58.1%) ulcers were on the right lower limb while 47 (34.5%) were on the left. Ten (7.4%) patients had ulcers on both feet. Multiple ulcers were seen on one foot in eight (5.9%) patients. The forefoot involving the toes was commonly affected in 60.3% of cases. Neuropathic ulcers were the most common type of DFU accounting for 57.4% of cases.

According to Wagner's classification (Table 2), Wagner's grade 4 and 5 ulcers (gangrenous diabetic foot ulcers) were the most prevalent at 29.4% and 23.5% respectively (Table 3). Patients with gangrenous diabetic foot ulcers (Wagner score ≥ 4) were significantly more likely to have delayed presentation to hospital than those with non- gangrenous diabetic foot ulcers (Wagner score < 4) (P = 0.021).

**Table 2:** Wagner's classification of diabetic foot ulcers

Ulcer grading	Description
Grade 0	No ulcer but high-risk foot
Grade 1	Superficial ulcer
Grade 2	Deep ulcer, no bony involvement or abscess
Grade 3	Abscess with bony involvement (as shown by X-ray)
Grade 4	Localized gangrene e.g. toe, heel etc
Grade 5	Extensive gangrene involving the whole foot

Note: Grade 1--3 ulcers are termed *non-gangrenous ulcers *and Grade 4 and 5 ulcers are termed *gangrenous ulcers*

**Table 3:** Clinical characteristics

Clinical characteristics	Frequency	Percentage
**Duration of diabetes (in years)**		
Newly diagnosed	38	27.9
< 1	14	10.3
1-5	32	25.5
>5	52	38.2
**Duration of DFU (in weeks)**		
<4	40	29.4
4-52	64	47.1
>52	32	23.5
**Type of Diabetes Mellitus**		
Type 1	2	1.5
Type 2	134	98.5
**Previous history of DFU**		
Yes	14	10.3
No	122	89.7
**Previous history of amputation**		
Yes	6	4.4
No	130	95.6
**Anatomical site**		
Forefoot	82	60.3
Midfoot	18	13.3
Hindfoot	10	7.4
Whole foot	26	19.1
**Foot affected**		
Right	79	58.1
Left	47	34.5
Both	10	7.4
**Type of ulcer**		
Neuropathic	78	57.4
Ischemic	42	30.8
Neuro-ischemic	6	4.4
Unclassified	10	7.4
**Wagner's classification**		
Stage 0	-	-
Stage 1	6	4.4
Stage 2	28	20.6
Stage 3	30	22.1
Stage 4	40	29.4
Stage 5	32	23.5

The majority of patients (98; 72.1%) were treated surgically and the remaining thirty-eight patients (27.9%) were treated conservatively with daily dressing and antibiotics. Most patients who were treated surgically underwent lower limb amputations in 56.7% of cases (Table 4). On stratification by severity of ulcers, patients with gangrenous DFU (Wagner score ≥ 4) were significantly more likely to have limb amputation than those with non-gangrenous DFU (Wagner score < 4) (P = 0.015).

**Table 4:** Type of operations performed (n = 127)

Type of operation	Frequency	Percentage
**Debridement**	**42**	**33.1**
**Lower limb amputation**	**72**	**56.7**
*Minor amputation*		
• Toe /Rye's amputation	37	51.4
*Major amputation*		
• Syme's amputation	5	6.9
• Below knee amputation	25	34.7
• Above knee amputation	5	6.9
**Skin grafting**	**6**	**4.7**
**Incision & drainage**	**5**	**3.9**
**Sequestrectomy**	**2**	**1.6**

A total of 64 post-operative complications were recorded in 46 (33.8%) of patients of which surgical site infection was the most common complication accounting for 18.8% of cases (Table 5). Complication rate was significantly high in patients who had major lower limb amputation than patients who had minor lower limb amputation (42.2% versus 16.7%) (P = 0.006)

**Table 5:** Post-operative complications (N = 64)

Complication	Frequency	Percentage
Surgical site infection	12	18.8
Revision amputation	11	17.2
Stump gangrene	9	14.1
Wound dehiscence	9	14.1
Phantom pain	7	10.9
Diabetic coma	7	10.9
Wound hematoma	5	7.8
Skin grafting failure	2	3.1
Anemia	2	3.1

Eight out of 12 (66.7%) cultured specimens had positive bacterial growth within 48 hours of incubation while 4 (33.3%) had negative bacterial growth. One out of 8 cultured specimens (12.5%) had pure bacterial growth while seven (87.5%) had polymicrobial bacterial growths. *Staphylococcus aureus *was the most frequent microorganism isolated 4 (50.0%), followed by *Escherichia coli *3 (37.5%) and *Klebsiella pneumoniae *2; (25.0%). *Pseudomonas spp *and *Proteus spp *were the least bacteria isolated. Anaerobic cultures were not performed. Antibacterial susceptibility testing revealed that most of pathogens isolates (i.e. *Staphylococcus aureus, Escherichia coli, Klebsiella pneumoniae, Pseudomonas spp *and *Proteus spp) *had multiple resistant to almost all tested antibiotics (such as ampicillin, augumentin, cotrimoxazole, tetracycline, penicillin, gentamicin, erythromycin, oxacillin etc). The majority of isolates were sensitive to meropenem (100%), imipenem (100%), vancomycin (81.8%), clindamycin (55.6%) and Ciprofloxacillin (53.6%). Table 6 shows the relationship between surgical procedures performed and postoperative complications.

**Table 6:** Surgical procedure versus Post-operative complications

Complications	Surgical procedures					Total
	Debridement	Lower limb amputation	Skin grafting	Incision & Drainage	Sequestrectomy	
SSI	4(33.3%)	3 (25.0%)	1(8.3%)	3(25.0%)	1 (8.3%)	12 (100%)
Rev. amputation	-	11(100%)	-	-	-	11(100%)
Stump gangrene	-	9 (100%)	-	-	-	9(100%)
Wound dehiscence	-	9 (100%)	-	-	-	9(100%)
Phantom pain	-	7(100%)	-	-	-	7(100%)
Wound hematoma	-	5(100%)	-	-	-	5(100%)
SG failure	-		2 (100%)	-	-	2 (100%)
Anemia	-	2 (100%)	-	-	-	2(100%)
Diabetic coma	2(28.6%)	1 (14.3%)	2 (28.6%)	2 (28.6%)	-	7(100%)

Keys: SSI = Surgical site infection, Rev. amputation = Revision amputation, SG failure = Skin grafting failure

The mean length of hospital stay was 36.24 ± 12.62 days (ranged 18-128 days). Patients who developed postoperative complications stayed long in the hospital (P = 0.012). Eighteen patients died giving a mortality rate of 13.2%. Mortality was significantly related to complications of diabetes mellitus (P = 0.002) and Wagner's grade ≥ 4 (P = 0.011). The causes of death were sepsis in seven (38.9%) patients, diabetic coma in five (27.8%), hypertension in three (16.7%), renal failure in two (11.1%) and cardiac arrest in one (5.6%).

## Discussion

In this review, the prevalence of diabetic foot ulcers amongst diabetic patients at Bugando Medical Centre was 3.2% which is comparable to studies in Kenya and South Africa [5,13]. Studies in Netherlands and Iran found high prevalence of 20.0% and 20.4% respectively [6,9]. These differences in prevalence may be a reflection of regional variations in prevalence of diabetes mellitus and the local operating risk factors of diabetic foot ulcer disease. High prevalence of DFUs in developing countries like Tanzania is due to illiteracy, poor socioeconomic status, bare-foot walking and inadequate facilities for diabetes care.

In our study, males were more affected than females with a male to female ratio of 1.2:1 which is in agreement with other studies [2,5,14]. Male predominance may be attributed to their smoking habits which were recorded in 35.3% of cases (all of them were males). Smoking is a contributory factor as a result of vascular wall thickening, reduction in blood circulation and ischemic changes in the affected neurons [15]. The resultant effect is also loss of sensation and increased predisposition to injuries.

The mean age of the patients was 54.32 years which is comparable to other studies done elsewhere [2,5,14]. Morbach *et al *[16] compared foot disease in Germany, India and Tanzania and found that German patients were significantly older (70.5 years) compared with those from Tanzania (51.4 years) and India (56.4 years). These studies were conducted in different centers that offer diabetes care of different qualities. This comparable mean age may suggest certain time-dependent risk factors in the evolution and course of diabetic foot ulcer disease which are common to diabetes in whatever environment. Age of onset of diabetes is also different in continents.

In this study, the median duration of diabetes is in keeping with other studies [5,10,17]. Morbach *et al *[16] found a significantly long mean duration of diabetes among German (14.0 ± 10.8 years) and Indian (11.7 ± 7.1 years) patients than among Tanzanian patients (5.1 ±.4.8 years). This finding may imply the differences in the quality of diabetes care where German and Indian patients, on average have longer duration of diabetes exposure before they develop foot ulcers. It is possible that better diabetes care that they receive delays the onset of foot ulcer disease.

The majority of patients in the present study presented to the surgical department between four weeks and 52 weeks (median of 18 weeks) of onset of an ulcer. Similar observation was also reported by other studies [2,5,14]. Late presentation in our patients may be attributed to low socioeconomic status, poverty, lack of diabetes education (regarding the importance of general foot care, the significance of diabetes and its complications), unrecognized foot trauma from walking barefoot and lack of access to medical care. Other contributing factors for late presentation include attempts at home surgery, trust in faith healers and undetected diabetes.

Wagner's classification which is based on severity of diabetic foot is widely used by surgeons [18]. According to Wagner's classification our patients were in the severe forms as grades IV and V constituted 52.9% collectively, and this is similar to what has been reported in other studies with an incidence range from 42% to 68% [2,18,19], but still less than the 74% reported in a western Sudan study [14]. High percentages of advanced foot ulcer disease in our study may be related to duration of diabetes, late presentation to healthcare professionals and presence of co-morbidities.

Wagner's classification score may be different for a surgeon as compared to physicians in the internal medicine. Physicians in the internal medicine receive diabetic foot problem at an earlier stage as compared to surgeons in the surgical department, where patients are admitted at advanced stages. Some patients may report to surgeons directly but the vast majority of them are referred to surgeons by physicians from internal medicine or endocrinologists, as part of the combined management. This is evident from our study where most of patients presented to the surgical department with advanced disease (Wagner's grade IV-V). This could be a reflection of inadequate education on diabetic foot care. Diabetic foot ulcer is one of the preventable and curable complications of diabetes [18,19]. Physicians in internal medicine have an important role in the prevention, early diagnosis and management of diabetic foot complications. The physicians from internal medicine or endocrinologists should do risk assessment in order to determine early the presence of risk to the foot [19].

Diabetic foot ulcers constitute a major public health problem for diabetic patient in sub-Saharan Africa where more than half of all limb amputations are carried out in patients with diabetes mellitus [18].

The rate of lower limb amputations in our study was 56.7% which is higher than rates reported in other studies [2,10,18,20-22]. This high amputation rate in our study could be attributed to the late presentation and severity of the disease on presentation. It is clearly evident from our study that more than half of our patients presented with high Wagner's grade (≥ 4) which resulted in the high rate of amputation. In this study, patients who had major lower limb amputation had significantly high complication rate than patients who underwent minor lower limb amputation.

Measures such as strict glycaemic control as well as participation in multi-disciplinary diabetic clinics consisting of vascular, orthopedic surgeons, internist, podiatrist, rehabilitation physician, orthopaedic shoemaker, and diabetic specialist nurse have been shown to significantly reduce complications and amputation rates [4,11]. This is not the set up in our hospital. Using such an approach a 50-85% reduction in amputation has been described in some studies [4,18,19].

Diabetic foot infections are one of the major causes of morbidity and mortality, especially in developing countries like Tanzania due to illiteracy; poor socioeconomic status, bare-foot walking and inadequate facilities for diabetes care. In this study, surgical site infection was the most common postoperative complication accounting for 18.8% of cases. The bacteriological patterns revealed polymicrobial bacterial growths with *Staphylococcus aureus *and *Escherichia coli *predominating. This is consistent with reports from other studies [2,23-27]. Ati *et al *[28] reported a high frequency of monomicrobial bacterial infections.

All the microorganisms isolated in this study showed high resistance to commonly used antibiotics except for Meropenem and imipenem which were all 100% sensitive respectively. Unfortunately, these antibiotics are expensive for the level of economical development which subsists in this part of the developing world. The finding of polymicrobial infection and multiple resistant to commonly used antibiotics calls for immediately surgical intervention. Antibiotic susceptibility testing remains of paramount importance in the management of diabetic foot ulceration.

The mean duration of hospital stay in our study was 36.24 ± 12.62 days which is higher compared to what was reported in other studies [10,14]. This variation might be related to differences in hospital facilities, severity of illness and availability of outpatient supportive care.

The mortality in our study was found to be 13.2%, mainly in patients with severe sepsis presented as Wagner's Grade IV and V, which is higher than that reported in other series [2,14,18]. The reason for high mortality rate in our study can be explained by the fact that, some of the patients were admitted in our hospital with advanced DFUs and sepsis, leading to multiple organ failure and death. A study in Tanzania revealed that the overall mortality rates for amputees and non-amputees were similar (29%); the highest in-patient mortality rate (54%) was observed among patients with severe (Wagner grade ≥ 4) ulcers who did not undergo surgery. Thus mortality rates among patients with severe ulcers remain high despite surgery and surgery undertaken during the less severe stages of ulcers may improve patient outcome. Early recognition of lesions and prompt initiation of the appropriate antibiotic therapy, as well as aggressive surgical debridement of necrotic tissue and bones, and modification of host factors i.e. hyperglycemia, concomitant arterial insufficiencies are all equally important for successful outcome [29].

The limitation of the present study was that only patients who were referred to the surgical department from diabetic clinic or medical wards were included in the study, which is underestimation of the prevalence of DFUs in the hospital, as patients with diabetes are also routinely admitted to other departments of the hospital. However, despite this limitation, the study has highlighted our experiences in the surgical management of diabetic foot ulcers.

## Conclusion

Diabetic foot ulceration constitutes a major source of morbidity and mortality among patients with diabetes mellitus at Bugando Medical Centre and is the leading cause of non-traumatic lower limb amputation. A multidisciplinary team approach targeting at good glycaemic control, education on foot care and appropriate footware, control of infection and early surgical intervention is required in order to reduce the morbidity and mortality associated with DFUs.

## Competing interests

The authors declare that they have no competing interests.

## Authors' contributions

PLC - Study design, literature search, data analysis, manuscript writing, editing and submission of the manuscript, JBM, RMD, RK, HJ, MDM, JBK and NM participated in Study design, data analysis, manuscript writing & editing and JMG was involved in study design, data analysis, coordination and supervision of manuscript writing & editing. All the authors read and approved the final manuscript.
